# Urine Ammonium Concentrations and Cardiovascular and Kidney Outcomes in Systolic Blood Pressure Intervention Trial Participants with CKD

**DOI:** 10.34067/KID.0000000000000501

**Published:** 2024-07-22

**Authors:** Alexander L. Bullen, Ronit Katz, Jesse Seegmiller, Pranav S. Garimella, Simon B. Ascher, Dena E. Rifkin, Kalani L. Raphael, Michael G. Shlipak, Joachim H. Ix

**Affiliations:** 1 Nephrology Section, Veterans Affairs San Diego Healthcare System, La Jolla, California; 2 Division of Nephrology and Hypertension, Department of Medicine, University of California San Diego, San Diego, California; 3 Department of Obstetrics and Gynecology, University of Washington, Seattle, Washington; 4 Department of Laboratory Medicine and Pathology, University of Minnesota, Minneapolis, Minnesota; 5 Kidney Health Research Collaborative, Department of Medicine, University of California, San Francisco, California; 6 Division of Hospital Medicine, University of California Davis, Sacramento, California; 7 Division of Nephrology and Hypertension, Department of Internal Medicine, University of Utah, Salt Lake City, Utah; 8 VA Salt Lake City Health Care System, Salt Lake City, Utah; 9 Department of Medicine, San Francisco VA Medical Center, San Francisco, California

**Keywords:** acidosis, AKI, cardiovascular events, CKD, mortality, tubule cells, biomarkers

## Abstract

**Key Points:**

- Among nondiabetic individuals with hypertension and CKD, higher urine ammonium concentration is associated with higher risk of cardiovascular disease.
- Urine ammonium was not associated with all-cause mortality or CKD progression, AKI, or linear eGFR decline in the Systolic Blood Pressure Intervention Trial cohort.

**Background:**

Impaired urine ammonium excretion is common in CKD and may identify risk of metabolic acidosis earlier than reductions in serum bicarbonate or pH and thus may have associations with cardiovascular disease (CVD) outcomes. We evaluated the association of urine ammonium with CVD and kidney outcomes among persons with hypertension and nondiabetic CKD enrolled in a trial of BP reduction.

**Methods:**

We measured urine ammonium concentration in spot urine specimens collected at baseline among 2092 participants of the Systolic Blood Pressure Intervention Trial (SPRINT) with an eGFR <60 ml/min per 1.73 m^2^. We used multivariable-adjusted Cox models to evaluate associations of urine ammonium concentration with the SPRINT CVD composite outcome (myocardial infarction, acute coronary syndrome, stroke, heart failure, or CVD death), all-cause mortality, the SPRINT kidney composite outcome (50% kidney function decline, ESKD, or transplant), and AKI.

**Results:**

At baseline, the mean (SD) age was 73 (9) years; 40% were female; and 25% were Black participants. The mean (SD) serum bicarbonate was 25.6 (2.8) mmol/L, median (interquartile range) urine ammonium concentration was 14.4 (9.5–23.1) mmol/L, and median (interquartile range) eGFR was 49 (39–55) ml/min per 1.73 m^2^. There were 255 CVD composite events, 143 deaths, 63 kidney composite events, and 146 AKI events during a median follow-up of 3.8 years. In multivariable models, each two-fold higher urinary ammonium concentration was associated with a 26% (95% confidence interval, 1.05 to 1.52) higher risk of the CVD composite, whereas there was no association with all-cause mortality, the SPRINT kidney composite outcome, or AKI.

**Conclusions:**

Among nondiabetic individuals with hypertension and CKD, higher urine ammonium concentration is associated with higher risk of CVD. Further studies are needed to evaluate this association in other cohorts.

## Visual Abstract

**Figure:**
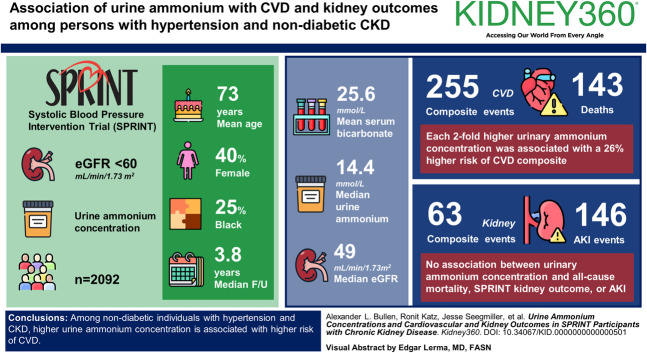

## Introduction

Metabolic acidosis is common among persons with advanced CKD^1,2^ and is associated with bone demineralization,^3^ CKD progression,^4^ and mortality.^5,6^ Metabolic acidosis is modifiable, which could theoretically prevent these complications. Yet acid-mediated organ injury occurs before overt acidosis develops that may be identified with serum bicarbonate, so reductions in serum bicarbonate may be late manifestations of this disease process.^7^ Thus, evaluating the association between other markers of acidosis and cardiovascular disease (CVD) and mortality is consequential.

The kidney tubules are central to homeostatic acid–base control using two key processes. They reabsorb the filtered load of bicarbonate, and they produce and excrete ammonium, which permits generation of new bicarbonate to replace any bicarbonate consumed by buffering.^8^ The standard clinical assessment consists solely of measuring serum total carbon dioxide (CO_2_) concentration; however, urinary ammonium excretion decreases before overt metabolic acidosis.^8^ In persons with advanced CKD and normal serum bicarbonate, lower urinary ammonium excretion is associated with faster decline in GFR and increased risk of mortality.^2^ However, there is conflicting evidence regarding whether high or low urine ammonium levels are associated with worse outcomes on the basis of human and animal data.^9–12^

In this study, we evaluated the associations of urine ammonium with subsequent risk of CVD outcomes, all-cause mortality, and kidney events among hypertensive persons with CKD in the Systolic Blood Pressure Intervention Trial (SPRINT). We have previously shown that impaired kidney tubular functions—assessed using other kidney function biomarkers—are associated with risk of CVD, kidney events, and mortality in SPRINT participants with CKD.^13–15^ *A priori*, we hypothesized that reduced urine ammonium may serve as an additional biomarker of abnormal kidney tubule function and may, therefore, be associated with similar outcomes.

## Methods

### Study Design and Participants

The design and primary results of SPRINT have been published previously.^16^ In brief, SPRINT was a clinical trial that randomized persons at high risk of CVD events to a systolic BP (SBP) target of <120 mm Hg (intensive) versus <140 mm Hg (standard). Inclusions required age 50 years and older, SBP between 130 and 180 mm Hg, and increased risk of CVD defined by prior clinical or subclinical CVD other than stroke, 10-year risk of CVD of ≥15% on the Framingham risk score, CKD defined as eGFR 20–59 ml/min per 1.73 m^2^, or age 75 years or older. Major exclusion criteria were diabetes mellitus, proteinuria >1 g/d, polycystic kidney disease, prior stroke or transient ischemic attack, symptomatic heart failure (HF), or a left ventricular ejection fraction <35%. A total of 9361 participants were randomized between November 2010 and March 2013. Participants were randomly assigned (1:1 ratio) to the two treatment arms. The antihypertensive regimens were adjusted to maintain SBPs according to the randomized treatment target. Participants attended visits monthly for the first 3 months and then every 3 months thereafter.^17^ Venous blood and urine specimens were processed immediately, shipped overnight on ice, and stored at −80°C at the central laboratory. All participants provided written informed consent. This study was approved by the committees on human research at the Veterans Affairs San Diego Healthcare System and University of California San Diego.

For this ancillary study, we measured urine ammonium concentrations from spot urine samples collected at baseline among 2514 SPRINT participants with a baseline eGFR <60 ml/min per 1.73 m^2^ by the combined CKD Epidemiology Collaboration equation for creatinine and cystatin C.^18^ Baseline serum bicarbonate was measured using an enzymatic method with phosphoenolpyruvate carboxylase on Roche CO_2_-L reagent or Roche Cobas 6000 Chemistry Analyzers (Roche Diagnostics Corporation) at the University of Minnesota Core Laboratory.^19^ We excluded 422 participants (17%) because of unavailable urine specimens, resulting in a final analytic sample of 2092 for this analysis. Of these, 1943 (92.9%) were fasting, 103 (5%) were not fasting, and 46 (2.2%) had missing or unknown fasting status at the time of urine collection.

### Exposure

Urine specimens were stored at −80°C until biomarker measurement, without prior thaw. Urine ammonium was measured in duplicate and the results were averaged at the University of Minnesota Advanced Research and Diagnostic Laboratory, the central laboratory for the SPRINT trial, using a glutamate dehydrogenase assay on a standard clinical chemistry analyzer with a coefficient of variation of 4.6%.

### Outcomes

Our primary outcome was the SPRINT primary composite CVD outcome, which included myocardial infarction (MI), acute coronary syndrome (ACS) not resulting in MI, stroke, acute decompensated HF, or death from cardiovascular causes. Secondary outcomes were all-cause mortality, the individual components of the composite CVD outcome, a composite kidney outcome (50% eGFR decline [confirmed by repeat testing ≥90 days later], or kidney failure requiring dialysis, or transplant), AKI, and annualized eGFR change, expressed as percentage change from baseline. Clinical events occurring during follow-up were ascertained primarily through surveillance of self-reported events obtained through structured interviews every 3 months and through laboratory and electrocardiogram data collected by the study and were adjudicated by members of the Morbidity and Mortality subcommittee masked to treatment assignment.^20^ Data on occurrence of AKI episodes were collected during safety monitoring for serious adverse events in SPRINT. Participants were considered to have AKI if, during a hospitalization, an AKI diagnosis was listed in the hospital discharge summary, and it was determined by the central SPRINT safety committee to be one of the top reasons for hospitalization or continued hospitalization. If available cases of AKI were noted in emergency department visits without subsequent hospitalization, these were also included in our analysis.

### Statistical Analyses

We used Spearman correlation coefficients to evaluate the correlation between urine ammonium/urine creatinine, serum bicarbonate, eGFR, and urine albumin-creatinine ratio at the baseline visit. We examined the cross-sectional associations of demographic and clinical risk factors with urine ammonium levels. Albuminuria was log-transformed because of right skewness. Model 1 included each variable individually. In model 2, all the variables were entered into the model, except for eGFR and albuminuria. eGFR and albuminuria were added in model 3. The reported results represent the percentage change in urine ammonium levels for a 1-unit increase in the respective variable, which was calculated as 100×*β* coefficient.

We then evaluated the association of urine ammonium with CVD, mortality, composite kidney outcome, and AKI using Cox proportional hazards models. We again log-base-2 transformed urine ammonium to facilitate interpretation of parameter estimates as “per two-fold higher.” To assess the functional form of associations, we also evaluate quartiles of urine ammonium concentration, setting the lowest as the reference category. Because urine creatinine is susceptible to bias by muscle mass and hydration status at the time of urine collection, urine ammonium was analyzed without indexing. Instead, we adjusted for urine creatinine in the multivariable models to correct for urine tonicity at the time of urine specimen collection.^21^ Covariates for multivariable models were selected *a priori* on the basis of biological plausibility. Model 1 was adjusted for age, sex, race, urine creatinine, and randomization arm. Baseline serum potassium, SBP, diastolic BP, number of antihypertensives medications, angiotensin-converting enzyme inhibitor or angiotensin II receptor blocker use, diuretic use, history of CVD or HF, and tobacco use at the time of the baseline visit, concurrent with urine ammonium measurement, were added in model 2. Baseline eGFR and urine albumin were additionally included in model 3. Serum bicarbonate was added in model 4.

We tested for interactions between urine ammonium, CVD, and mortality by sex, fasting status at the baseline visit, baseline diuretic use, and randomized treatment arm.

We evaluated the nonlinear relationship between urine ammonium and CVD using restricted cubic spline with a model adjusted for age, sex, race, urine creatinine, randomization arm, baseline eGFR, and urine creatinine.

In sensitivity analyses, we evaluated the association between urine ammonium and CVD and mortality among participants with prevalent metabolic acidosis (defined as a baseline CO_2_ <22 mmol/L) and according to baseline eGFR (<45 versus ≥45 ml/min per 1.73 m^2^) using the covariates from model 3.

We used linear mixed models to evaluate the association of urine ammonium with annualized eGFR change. Model 1 was adjusted for age, sex, race, urine creatinine, and randomization arm. SBP, diastolic BP, number of antihypertensives medications, angiotensin-converting enzyme inhibitor or angiotensin II receptor blocker use, diuretic use, history of CVD or HF, and tobacco use were added in model 2. Baseline eGFR and urine albumin-creatinine ratio were additionally included in model 3. Serum bicarbonate was added in model 4. We also performed sensitivity analyses by randomization arm, using similar models.

All analyses were conducted using Stata/MP version 15.1 (StataCorp LLC, College Station, TX). *P* values <0.05 were considered statistically significant for all analyses including interaction terms.

## Results

### Baseline Characteristics

The study sample consisted of 2092 SPRINT participants with CKD at baseline; 50.5% were randomized to the intensive arm. Most of the participants (92.9%) were fasting before providing the urine sample (Supplemental Table 1), and only 12 (0.6%) were taking alkali therapy. The mean (SD) age was 73±9 years, 40% were female, and 25% were Black participants. The median (interquartile range [IQR]) eGFR at baseline was 49 (39–55) ml/min per 1.73 m^2^, and the median (IQR) urine albumin-creatinine ratio was 13 (7–43) mg/g. The median (IQR) urine ammonium concentration was 14.4 (9.5–23.1) mmol/L. Table 1 presents participant characteristics by quartiles of urine ammonium concentration. Compared with those in the lower quartiles, participants in the higher quartiles were younger, more frequently male, and current smokers; less likely to be using diuretics; and had higher mean body mass and eGFR and lower albuminuria at the baseline visit. Prevalence of CVD, randomization arm, serum bicarbonate, and SBP seemed similar across quartiles. When evaluating urine ammonium on a continuous scale and evaluating correlations, higher urine ammonium was weakly correlated with lower serum bicarbonate concentrations and higher eGFR, but had no correlation with urine albumin-creatinine ratio (Supplemental Table 2).

**Table 1:** Participant characteristics by urine ammonium excretion quartiles

Characteristics	NH4 Quartile 1	NH4 Quartile 2	NH4 Quartile 3	NH4 Quartile 4	Total
Range, mmol/L	<9.4	9.4–14.4	14.5–23.1	>23.1	
*N*	523	524	523	522	2092
Age, yr (SD)	74 (9)	74 (9)	73 (9)	71 (9)	73 (9)
Female, *n* (%)	250 (48)	211 (40)	189 (36)	178 (34)	828 (40)
**Race, *n* (%)**					
Non-Hispanic White	341 (65)	349 (67)	362 (69)	347 (67)	1399 (67)
Non-Hispanic Black	143 (27)	117 (22)	114 (22)	141 (27)	515 (25)
Hispanic and other	39 (8)	58 (11)	47 (9)	34 (7)	178 (9)
Intensive randomization arm, *n* (%)	257 (49)	264 (50)	270 (52)	266 (51)	1057 (51)
History of cardiovascular disease, *n* (%)	134 (26)	125 (24)	155 (30)	127 (24)	541 (26)
**Smoking status, *n* (%)**					
Never	237 (45)	244 (47)	225 (43)	241 (46)	947 (45)
Former	248 (47)	240 (46)	249 (48)	226 (43)	963 (46)
Current	38 (7)	40 (8)	49 (9)	55 (11)	182 (9)
BMI, kg/m^2^, (SD)	29.0 (5.9)	29.2 (5.8)	29.7 (5.4)	30.5 (6.1)	29.6 (5.9)
Serum bicarbonate, mmol/L (SD)	25.9 (2.8)	25.7 (2.7)	25.6 (2.6)	25.2 (2.9)	25.6 (2.8)
Serum potassium, mmol/L (SD)	4.35 (0.43)	4.32 (0.48)	4.31 (0.46)	4.22 (0.49)	4.30 (0.47)
Total cholesterol, mg/dl (SD)	183 (39)	187 (42)	180 (41)	181 (40)	183 (41)
High-density lipoprotein, mg/dl (SD)	53 (14)	53 (15)	51 (14)	51 (14)	52 (14)
Mean SBP, mm Hg (SD)	141 (17)	140 (16)	138 (15)	139 (16)	139 (16)
Mean DBP, mm Hg (SD)	74 (12)	74 (12)	74 (13)	76 (12)	75 (12)
Use of ACEi or ARBs, *n* (%)	320 (61)	340 (65)	312 (60)	322 (62)	1294 (62)
Use of diuretics, *n* (%)	276 (53)	301 (57)	272 (52)	252 (48)	1101 (53)
Median eGFR, (IQR)	46 (35–53)	48 (38–55)	49 (41–55)	51 (43–56)	49 (39–55)
Median urine ACR, mg/g (IQR)	17 (7–77)	14 (7–56)	11 (6–29)	12 (6–37)	13 (7–43)

ACEi, angiotensin-converting enzyme inhibitor; ACR, albumin-creatinine ratio; ARB, angiotensin II receptor blocker; BMI, body mass index; DBP, diastolic BP; IQR, interquartile range; SBP, systolic BP.

Table 2 shows the cross-sectional association of baseline demographic and clinical variables with urine ammonium. Male sex, higher body mass index, and higher baseline eGFR were directly associated with higher urine ammonium levels, whereas age, serum bicarbonate level, serum potassium, and albuminuria were associated with lower urine ammonium levels independent of all other covariates.

**Table 2:** Cross-sectional association of urine ammonium with clinical characteristics of Systolic Blood Pressure Intervention Trial participants with CKD

Characteristic	Model 1^a^		Model 2^b^		Model 3^c^	
	% (95% CI)	*P* Value	% (95% CI)	*P* Value	% (95% CI)	*P* Value
Age (per SD=9)	−8 (−10 to −5)	<0.001	−4 (−8 to −1)	0.015	−3 (−7 to −0.1)	0.048
Male (female=ref)	13 (8,18)	<0.001	13 (7 to 19)	<0.001	11 (5 to 17)	<0.001
**Race to *n* (%)**						
Non-Hispanic White	0 (ref)		0 (ref)		0 (ref)	
Non-Hispanic Black	1 (−5 to 7)	0.763	−3 (−10 to 3)	0.352	−0.4 (−7 to 6)	0.914
Hispanic and other	−2 (−12 to 7)	0.616	−2 (−12 to 8)	0.681	−0.6 (−10 to 9)	0.897
Intensive randomization arm	1 (−4 to 6)	0.656	0.4 (−5 to 6)	0.889	−0.1 (−5 to 5)	0.964
History of cardiovascular disease	2 (−4 to 8)	0.564	2 (−5 to 8)	0.611	3 (−3 to 9)	0.385
**Smoking status**						
Never	0 (ref)		0 (ref)		0 (ref)	
Former	−3 (−8 to 3)	0.337	−3 (−9 to 3)	0.291	−2 (−8 to 3)	0.459
Current	11 (2 to 20)	0.023	4 (−6 to 13)	0.463	5 (−5 to 15)	0.288
BMI, kg/m^2^, (per SD=5.9)	6 (4 to 9)	<0.001	5 (2 to 8)	<0.001	5 (2 to 8)	<0.001
Serum bicarbonate, mmol/L (per SD=2.8)	−6 (−8 to −3)	<0.001	−4 (−7 to −2)	0.002	−8 (−22 to −5)	<0.001
Total cholesterol, mg/dl (per SD=41)	−2 (−5 to 0.1)	0.097	−1 (−4 to 2)	0.651	−1 (−4 to 2)	0.506
High-density lipoprotein, mg/dl (per SD=14)	−5 (−7 to −2)	<0.001	0.3 (−3 to 3)	0.852	−0.1 (−3 to 3)	0.928
SBP, mm Hg (per SD=16)	−3 (−6 to −1)	0.011	−4 (−7 to −1)	0.027	−2 (−5 to 1)	0.175
DBP, mm Hg (per SD=12)	5 (2 to 7)	<0.001	3 (−0.1 to 7)	0.055	3 (−1 to 6)	0.117
Use of ACEi or ARBs	−1 (−7 to 4)	0.682	−3 (−8 to 3)	0.32	−3 (−9 to 2)	0.228
Use of diuretics	−5 (−10 to 5)	0.074	−4 (−10 to 1)	0.12	−3 (−9 to 2)	0.238
Serum potassium, mmol/L (per SD=0.47)	−6 (−8 to −3)	<0.001	−7 (−10 to −5)	<0.001	−6 (−8 to −3)	<0.001
eGFR (per SD=10)	11 (9 to 14)	<0.001	—	—	10 (7 to 13)	<0.001
Urine ACR (per SD of log transformed ACR=1.45)	−8 (−11 to −6)	<0.001	—	—	−6 (−9 to −4)	<0.001

ACEi, angiotensin-converting enzyme inhibitor; ACR, albumin-creatinine ratio; ARB, angiotensin II receptor blocker; BMI, body mass index; CI, confidence interval; DBP, diastolic BP; SBP, systolic BP.

a Model 1: each variable entered into the model individually.

b Model 2: all variables entered into the model, except baseline eGFR and urine ACR.

c Model 3: all variables entered into the model, including eGFR and urine ACR.

### Association of Urine Ammonium with CVD Events and All-Cause Mortality

During a median follow-up of 3.8 years, there were 255 composite CVD events (incidence rate 3.4% per year). Each two-fold higher urine ammonium concentration was associated with a 20% (hazard ratio, 1.20; 95% confidence interval, 1.01 to 1.44) greater risk of the composite CVD end point in models that were adjusted for age, sex, race, randomization arm, and urine creatinine. When adding CVD risk factors (model 2) and when additionally adjusting for eGFR, albuminuria (model 3), and serum bicarbonate (model 4), this association was minimally altered, such that each two-fold higher urine ammonium concentration remained associated with a statistically significant 26% higher risk of CVD events in the final model (Table 3). Compared with the lowest quartile, the risk of CVD was higher in all other quartiles and statistically significant in the third quartile. Associations of urine ammonium with CVD events were similar irrespective of sex, fasting status, diuretic use, and randomization arm (*P*-interactions all ≥0.08).

**Table 3:** Association of urine ammonium with composite cardiovascular events in Systolic Blood Pressure Intervention Trial participants with CKD

Urine Ammonium	*N*	No. of Events	Incidence Rate, %/yr	Model 1^a^	Model 2^b^	Model 3^c^	Model 4^d^
				HR (95% CI)	HR (95% CI)	HR (95% CI)	HR (95% CI)
**CVD primary SPRINT event**							
Continuous (per doubling)	2092	255	3.41	1.20 (1.01 to 1.44)	1.20 (1.00 to 1.45)	1.26 (1.05 to 1.51)	1.26 (1.05 to 1.52)
Quartiles, mmol/L							
*<9.4*	523	56	2.99	1.00 (ref)	1.00 (ref)	1.00 (ref)	1.00 (ref)
*9.4–14.4*	524	70	3.77	1.36 (0.94 to 1.96)	1.34 (0.93 to 1.94)	1.38 (0.95 to 2.01)	1.38 (0.95 to 2.01)
*14.5–23.1*	523	75	4.03	1.59 (1.07 to 2.36)	1.57 (1.06 to 2.34)	1.75 (1.17 to 2.61)	1.75 (1.17 to 2.62)
*>23.1*	522	54	2.86	1.29 (0.80 to 2.06)	1.30 (0.81 to 2.08)	1.42 (0.87 to 2.29)	1.42 (0.87 to 2.31)
**All-cause mortality**							
Continuous (per doubling)	2092	143	2.1	1.00 (0.78 to 1.27)	0.97 (0.76 to 1.24)	1.04 (0.81 to 1.33)	1.01 (0.78 to 1.29)
Quartiles, mmol/L							
*<9.4*	523	43	2.56	1.00 (ref)	1.00 (ref)	1.00 (ref)	1.00 (ref)
*9.4–14.4*	524	28	1.66	0.68 (0.42 to 1.12)	0.68 (0.41 to 1.12)	0.72 (0.44 to 1.20)	0.70 (0.42 to 1.16)
*14.5–23.1*	523	38	2.21	0.98 (0.60 to 1.62)	0.96 (0.58 to 1.57)	1.13 (0.68 to 1.88)	1.09 (0.65 to 1.81)
*>23.1*	522	34	1.98	1.00 (0.56 to 1.80)	0.98 (0.55 to 1.75)	1.12 (0.62 to 2.02)	1.03 (0.57 to 1.88)

CI, confidence interval; CVD, cardiovascular disease; HR, hazard ratio; SPRINT, Systolic Blood Pressure Intervention Trial.

a Model 1: age, sex, race, urine creatinine, and randomization arm.

b Model 2: model 1+systolic BP, diastolic BP, number of antihypertensive meds, angiotensin-converting enzyme inhibitor or angiotensin II receptor blocker use, diuretic use, history of cardiovascular disease or heart failure, tobacco use, body mass index, LDL, and total cholesterol.

c Model 3: model 2+baseline eGFR and urine albumin.

d Model 4: model 3+serum bicarbonate.

Table 4 presents the association of urine ammonium with the individual components of the composite primary end point. Urine ammonium was more strongly associated with HF, ACS, and stroke, compared with CVD death and MI, although these associations did not reach statistical significance individually (Table 4).

**Table 4:** Association of urine ammonium with individual cardiovascular endpoints after multivariable adjustment

Urine Ammonium	HF	CVD Death	MI	ACS	Stroke
*N*	2092	2092	2092	2092	2092
Events	67	30	89	19	50
Incidence rate/100PY	0.90	0.40	1.19	0.25	0.67
Urine ammonium HR per two-fold higher (95% CI)	1.28 (0.89 to 1.82)	1.02 (0.60 to 1.76)	1.16 (0.84 to 1.59)	1.40 (0.67 to 2.93)	1.31 (0.88 to 1.95)

ACS, acute coronary syndrome; CI, confidence interval; CVD, cardiovascular disease; HF, heart failure; HR, hazard ratio; MI, myocardial infarctions.

Models adjusted for age, sex, race, urine creatinine, randomization arm, systolic BP, diastolic BP, number of antihypertensive meds, angiotensin-converting enzyme inhibitor or angiotensin II receptor blocker use, diuretic use, history of cardiovascular disease and heart failure, tobacco use, baseline serum potassium, baseline eGFR, and urine albumin.

**Figure 1:**
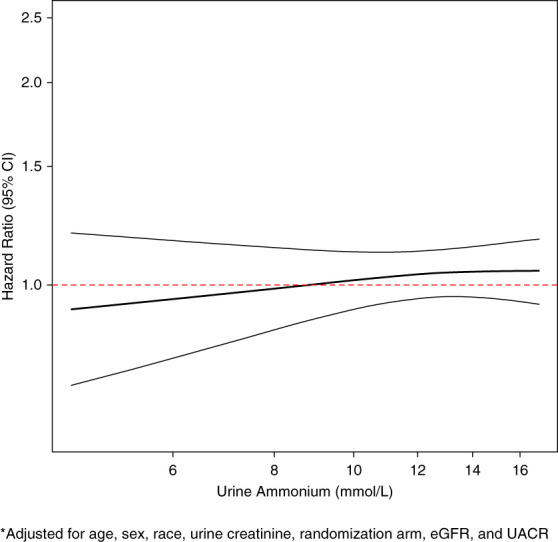
Association of baseline urine ammonium with CVD using restricted cubic spline.* *Adjusted for age, sex, race, urine creatinine, randomization arm, eGFR, and UACR. CVD, cardiovascular disease; UACR, urine albumin-to-creatinine ratio.

Because the association between urine ammonium levels and CVD was not monotonic across quartiles, we explored the nonlinear relationship using restrictive cubic spline. Higher urine ammonium level was associated with an incrementally higher risk of CVD in this analysis (Figure 1).

There were 143 deaths from any cause over the follow-up period (incidence 2.1% per year). In contrast to the models for CVD, the association of urine ammonium with mortality risk was near unity across the sequence of models (Table 3). Associations of urine ammonium with mortality were similar irrespective of sex, fasting status, diuretic use, or randomization arm (*P* interactions all ≥0.45).

In sensitivity analyses, the association of urine ammonium with CVD events and mortality was similar irrespective of baseline total CO_2_ and eGFR (*P* for interaction >0.2, Supplemental Tables 3 and 4, respectively).

### Association of Urine Ammonium with Kidney Disease Outcomes

There were 63 composite kidney events: 23 occurred in the standard arm and 40 in the intensive arm. When modeled continuously or in quartiles, urine ammonium was not associated with the composite kidney outcome (Table 5).

**Table 5:** Association of urine ammonium with the composite kidney outcome and AKI among Systolic Blood Pressure Intervention Trial participants with CKD

Urine Ammonium	*N*	No. of Events	Incidence Rate, %/yr	Model 1^a^	Model 2^b^	Model 3^c^	Model 4^d^
				HR (95% CI)	HR (95% CI)	HR (95% CI)	HR (95% CI)
**50% kidney function decline or kidney failure or transplant**							
Continuous (per doubling)	2092	63	0.94	1.07 (0.74 to 1.56)	1.03 (0.74 to 1.49)	1.24 (0.86 to 1.78)	1.15 (0.79 to 1.68)
Quartiles							
*<9.4*	523	22	1.33	1.00 (ref)	1.00 (ref)	1.00 (ref)	1.00 (ref)
*9.4–14.4*	524	15	0.90	0.89 (0.44 to 1.80)	0.91 (0.45 to 1.85)	0.90 (0.44 to 1.86)	0.85 (0.41 to 1.77)
*14.4–23.1*	523	10	0.59	0.72 (0.31 to 1.69)	0.74 (0.32 to 1.73)	1.09 (0.45 to 2.63)	0.99 (0.41 to 2.42)
*>23.1*	522	16	0.95	1.47 (0.62 to 3.47)	1.44 (0.62 to 3.36)	2.05 (0.85 to 5.00)	1.73 (0.70 to 4.32)
**AKI**							
Continuous (per doubling)	2092	146	1.90	1.11 (0.87 to 1.41)	1.10 (0.86 to 1.40)	1.21 (0.95 to 1.54)	1.23 (0.97 to 1.57)
Quartiles							
*<9.4*	523	36	1.89	1.00 (ref)	1.00 (ref)	1.00 (ref)	1.00 (ref)
*9.4–14.4*	524	34	1.78	1.01 (0.62 to 1.64)	1.02 (0.62 to 1.66)	1.13 (0.69 to 1.86)	1.15 (0.70 to 1.89)
*14.4–23.1*	523	37	1.91	1.12 (0.67 to 1.88)	1.13 (0.67 to 1.90)	1.43 (0.84 to 2.44)	1.46 (0.86 to 2.50)
*>23.1*	522	39	2.01	1.25 (0.69 to 2.24)	1.25 (0.69 to 2.26)	1.58 (0.87 to 2.88)	1.64 (0.89 to 3.02)

CI, confidence interval; HR, hazard ratio.

a Model 1: age, sex, race, urine creatinine, and randomization arm.

b Model 2: model 1+systolic BP, diastolic BP, number of antihypertensive meds, angiotensin-converting enzyme inhibitor or angiotensin II receptor blocker use, diuretic use, history of cardiovascular disease or heart failure, and tobacco use.

c Model 3: model 2+baseline eGFR and urine albumin.

d Model 4: model 3+serum bicarbonate.

There were 146 AKI events (59 in the standard arm and 87 in the intensive arm) over the follow-up period. As with the kidney disease composite, there was no association of urine ammonium with risk of AKI (Table 5).

Participants underwent a mean (SD) of 8.7±2.8 eGFR assessments. Higher urine ammonium was not significantly associated with eGFR decline across the sequence of models (Table 6). The association between urine ammonium with eGFR decline was similar by randomization arm (Supplemental Table 5).

**Table 6:** Association of urine ammonium with eGFR change (positive values=increase in eGFR slope, negative values=decline in eGFR slope)

Urine Ammonium	*N*	Mean eGFR Change, %/yr	Model 1^a^	Model 2^b^	Model 3^c^	Model 4^d^
			*β* (95% CI)	*β* (95% CI)	*β* (95% CI)	*β* (95% CI)
Continuous (per doubling)	2092	0.15 (−0.04 to 0.33)	0.04 (−0.20 to 0.28)	0.06 (−0.18 to 0.30)	0.05 (−0.19 to 0.29)	0.02 (−0.21 to 0.26)
**Quartiles (mmol/L)**						
<9.4	523	−1.42 (−1.74 to −1.10)	0 (ref)	0 (ref)	0 (ref)	0 (ref)
9.4–14.4	524	−1.18 (−1.50 to −0.86)	0.19 (−0.29 to 0.67)	0.17 (−0.31 to 0.65)	0.21 (−0.26 to 0.69)	0.18 (−0.30 to 0.66)
14.5–23.1	523	−1.04 (−1.35 to −0.72)	0.09 (−0.42 to 0.61)	0.03 (−0.49 to 0.54)	−0.18 (−0.69 to 0.33)	−0.23 (−0.74 to 0.28)
>23.1	522	−1.42 (−1.73 to −1.10)	−0.41 (−0.99 to 0.17)	−0.33 (−0.91 to 0.24)	−0.30 (−0.87 to 0.27)	−0.38 (−0.96 to 0.20)

CI, confidence interval.

a Model 1: age, sex, race, urine creatinine, and randomization arm.

b Model 2: model 1+systolic BP, diastolic BP, number of antihypertensive meds, angiotensin-converting enzyme inhibitor or angiotensin II receptor blocker use, diuretic use, history of cardiovascular disease or heart failure, and tobacco use.

c Model 3: model 2+baseline eGFR and urine albumin.

d Model 4: model 3+serum bicarbonate.

## Discussion

CVD is the main cause of morbidity and mortality among persons with CKD. In previous studies, we demonstrated that markers of kidney tubule dysfunction are independently associated with higher CVD risk above and beyond eGFR and albuminuria; thus, we hypothesized that lower urine ammonium would associate with higher risk of CVD.^14^ Contrary to our hypothesis, we observed that higher urine ammonium concentrations were associated with greater CVD risk among a large sample of hypertensive participants with CKD from SPRINT. This association seemed to be driven primarily by relations of urine ammonium with HF, ACS, and stroke. We found urine ammonium was not associated with all-cause mortality or CKD progression, AKI, or linear eGFR decline.

To our knowledge, no prior study has evaluated the relationship of urine ammonium with CVD risk. Several prior studies that evaluated associations of serum bicarbonate concentrations with CVD showed conflicting results. In the *post hoc* analysis of the Reduction of Endpoints in NIDDM with the Angiotensin II Antagonist Losartan trial and the Irbesartan Diabetic Nephropathy Trial, there were no significant associations between serum bicarbonate and CVD events.^22^ By contrast, in another study evaluating over 50,000 patients with CKD over a 10-year period, lower serum bicarbonate concentrations were associated with increased risk of CVD after adjusting for key covariates, including baseline eGFR.^23^ Among persons with metabolic acidosis, each 1 mEq/L lower serum bicarbonate was associated with greater incidence of HF, stroke, MI, and CVD death in another study.^23^ Similarly, in a study previously conducted among SPRINT participants with and without CKD at baseline, lower serum bicarbonate levels were associated with a higher CVD risk.^19^

There may be several explanations as to why two markers of acidosis seem to have discordant associations with CVD. One possibility is that total CO_2_ reflects more than acid/base status, including use of certain antihypertensive medications, hydration status, and pulmonary disease processes. Ammonium may also reflect other processes. It is widely believed that one of the main contributors for the development of CVD is cardiovascular calcification secondary to altered bone and mineral metabolism in patients with CKD.^24,25^ Acidic pH increases solubility of calcium, increases osteoclast activity, and suppresses bone formation by osteoblasts, resulting in bone loss.^26^ Through similar mechanisms, it may prevent calcification in the vasculature.^27^ Thus, it is possible that higher urine ammonium may associate with greater risk and burden of cardiovascular calcifications and may be associated with higher CVD event risk through this mechanism. Unfortunately, data on vascular calcification are not available in SPRINT, so this interesting hypothesis requires future study. We adjusted for potential confounders, including serum potassium, because it is inversely correlated with ammonia production.^28,29^ However, there remains a possibility of residual confounding.

Despite observing that urine ammonium is associated with CVD risk, we did not find an association of urine ammonium with mortality risk. Among participants with CKD in the African American Study of Kidney Disease and Hypertension (AASK), there was an association of lower urine ammonium with a composite outcome of death or kidney failure; however, when death was evaluated independently, no association was observed.^7^ In the NephroTest cohort, findings were similar.^30^ There are some key differences between the present study and the ones mentioned above. SPRINT specifically sought to recruit older participants at high risk of CVD. Among the SPRINT cohort with CKD, the mean age was over 15 years older in AASK and NephroTest. In addition, AASK only recruited Black patients with hypertensive CKD and NephroTest consisted of a European prospective hospital-based cohort. However, despite the heterogeneity among the populations, the studies have been consistent in their reporting of the absence of an association between urine ammonium and mortality.

In the Nephrotest cohort, those in the lowest tertile of urine ammonium excretion had higher odds of GFR decline and higher odds of kidney failure.^30^ In AASK, the low urine ammonium tertile was associated with 50% higher risk of death or kidney failure compared with participants in the highest tertile.^7^ In SPRINT, we did not find an association of urine ammonium with the composite kidney outcome, AKI, or linear eGFR decline. There are some key differences between our study and the ones previously mentioned. SPRINT participants had a higher baseline eGFR, and there was a small number of participants who progressed to ESKD in contrast to the Nephrotest study, where most of the patients had GFR between 15 and 44 ml/min per 1.73 m^2^, and approximately 19% of participants developed ESKD.^30^ In AASK, 16% of participants had CKD 4 or 5 and almost 30% of participants reached the composite outcome of death or ESKD.

This study has several strengths. We evaluated a well-characterized, large, and diverse sample of persons with CKD. CVD events were centrally adjudicated according to prespecified protocols by a panel of experts. Baseline urine ammonium was measured in duplicate to improve precision, and data were available from a wide variety of covariates, including eGFR and albuminuria, medication use, and fasting status. Multiple eGFRs were measured during follow-up on the basis of protocolized time points rather than by clinical indications, and AKI events were also centrally collected and adjudicated.

The study also has important limitations. SPRINT excluded persons with diabetes mellitus, stroke, or proteinuria >1 g/d, and the results may not generalize to populations with these conditions. Urine ammonium was only measured at baseline, so we were not able to assess variability of urine ammonium concentration over time in this study, nor the associations of change in ammonium with subsequent events. We did not have the systemic pH. We did not have information about the diet, so are unable to assess the dietary acid load. We measured urine ammonium in spot specimens. While urine ammonium may fluctuate throughout the day, prior studies have demonstrated similar relationships of urine ammonia with CKD progression and mortality irrespective of use of timed or spot urine specimens.^30^ Finally, we had a limited sample size, and we have not validated these results in an external cohort.

In summary, among a large cohort of trial participants with hypertension and CKD, higher urine ammonium was associated with CVD events, but not mortality. As prior studies demonstrate that lower urine ammonium is associated with CKD progression, these findings suggest that urine ammonium may reflect more than tubule dysfunction and may implicate unique biological pathways linked with CKD progression versus CVD events. Future clinical trials of alkali therapy—a key driver of urine ammonium concentrations^31^ —should pay close attention to CVD risk beyond CKD progression.

## Supplementary Material

## Data Availability

All data are included in the manuscript and/or supporting information.

## Supplemental Material

Supplemental Table 1. Association of eGFR, urine ammonium, and serum bicarbonate with baseline fasting status.

Supplemental Table 2. Correlation matrix of key variables relative to spot urine ammonium concentrations among SPRINT participants with CKD.

Supplemental Table 3. Association of baseline urine ammonium with primary CVD event and all-cause mortality in SPRINT participants by metabolic acidosis status at baseline.

Supplemental Table 4. Association of baseline urine ammonium with primary CVD event and all-cause mortality in SPRINT participants by eGFR at baseline.

Supplemental Table 5. Association of baseline urine ammonium with annualized eGFR change among SPRINT participants with CKD by randomization arm.
