# Supplementary material for: Urine Ammonium Concentrations and Cardiovascular and Kidney Outcomes in Systolic Blood Pressure Intervention Trial Participants with CKD
Source: Kidney360. 2024 Jul 22;5(8):1077–86. doi: 10.34067/KID.0000000000000501 (PMC11371348; doi:10.34067/KID.0000000000000501)
Supplement: Supplementary file 1 [file kidney360-5-1077-s001.pdf]

ASN American Society of Nephrology | JASN Journal of the American Society of Nephrology | KIDNEY360 Accessing Our World From Every Angle | CJASN Clinical Journal of the American Society of Nephrology

ASN Journal Disclosure Form

As per ASN journal policy, I have disclosed any financial relationships or commitments I have held in the past 36 months as included below. I have listed my Current Employer below to indicate there is a relationship requiring disclosure. If no relationship exists, my Current Employer is not listed.

S. Ascher reports the following:
Employer: UC Davis

I understand that the information above will be published within the journal article, if accepted, and that failure to comply and/or to accurately and completely report the potential financial conflicts of interest could lead to the following: 1) Prior to publication, article rejection, or 2) Post-publication, sanctions ranging from, but not limited to, issuing a correction, reporting the inaccurate information to the authors' institution, banning authors from submitting work to ASN journals for varying lengths of time, and/or retraction of the published work.

Name: Simon Ascher
Manuscript ID: K360-2024-000146R1
Manuscript Title: Urine ammonium concentrations and cardiovascular and kidney outcomes in SPRINT participants with Chronic Kidney Disease
Date of Completion: April 19, 2024
Disclosure Updated Date: April 19, 2024

ASN
American Society of Nephrology
JASN
Journal of the American Society of Nephrology
KIDNEY360
Accessing Our World From Every Angle
CJASN
Clinical Journal of the American Society of Nephrology

ASN Journal Disclosure Form

As per ASN journal policy, I have disclosed any financial relationships or commitments I have held in the past 36 months as included below. I have listed my Current Employer below to indicate there is a relationship requiring disclosure. If no relationship exists, my Current Employer is not listed.

A. Bullen reports the following:
Employer: VA San Diego Healthcare System; UC San Diego

I understand that the information above will be published within the journal article, if accepted, and that failure to comply and/or to accurately and completely report the potential financial conflicts of interest could lead to the following: 1) Prior to publication, article rejection, or 2) Post-publication, sanctions ranging from, but not limited to, issuing a correction, reporting the inaccurate information to the authors' institution, banning authors from submitting work to ASN journals for varying lengths of time, and/or retraction of the published work.

Name: Alexander L. Bullen
Manuscript ID: K360-2024-000146R2
Manuscript Title: Urine ammonium concentrations and cardiovascular and kidney outcomes in SPRINT participants with Chronic Kidney Disease
Date of Completion: June 20, 2024
Disclosure Updated Date: May 13, 2024

ASN American Society of Nephrology
JASN Journal of the American Society of Nephrology
KIDNEY360 Accessing Our World From Every Angle
CJASN Clinical Journal of the American Society of Nephrology

ASN Journal Disclosure Form

As per ASN journal policy, I have disclosed any financial relationships or commitments I have held in the past 36 months as included below. I have listed my Current Employer below to indicate there is a relationship requiring disclosure. If no relationship exists, my Current Employer is not listed.

P. Garimella reports the following:
Employer: UCSD; Honoraria: DCI Inc; Advisory or Leadership Role: Co Chair PKD Foundation CoE Advisory Board; Associate Editor BMC Nephrology; Editorial Board Member, Kidney Medicine; and Speakers Bureau: Otsuka.

I understand that the information above will be published within the journal article, if accepted, and that failure to comply and/or to accurately and completely report the potential financial conflicts of interest could lead to the following: 1) Prior to publication, article rejection, or 2) Post-publication, sanctions ranging from, but not limited to, issuing a correction, reporting the inaccurate information to the authors' institution, banning authors from submitting work to ASN journals for varying lengths of time, and/or retraction of the published work.

Name: Pranav S. Garimella
Manuscript ID: K360-2024-000146R1
Manuscript Title: Urine ammonium concentrations and cardiovascular and kidney outcomes in SPRINT participants with Chronic Kidney Disease
Date of Completion: April 18, 2024
Disclosure Updated Date: April 3, 2024

ASN Journal Disclosure Form

As per ASN journal policy, I have disclosed any financial relationships or commitments I have held in the past 36 months as included below. I have listed my Current Employer below to indicate there is a relationship requiring disclosure. If no relationship exists, my Current Employer is not listed.

J. Ix reports the following:
Employer: UCSD; Consultancy: AstraZeneca, Bayer, Jnana, Marius; Research Funding: Juvenile Diabetes Research Foundation; Honoraria: AstraZeneca, Bayer, Jnana, Marius; Advisory or Leadership Role: AlphaYoung; and Other Interests or Relationships: Executive Board for Kidney Disease: Improving Global Outcomes (KDIGO) -.

I understand that the information above will be published within the journal article, if accepted, and that failure to comply and/or to accurately and completely report the potential financial conflicts of interest could lead to the following: 1) Prior to publication, article rejection, or 2) Post-publication, sanctions ranging from, but not limited to, issuing a correction, reporting the inaccurate information to the authors' institution, banning authors from submitting work to ASN journals for varying lengths of time, and/or retraction of the published work.

Name: Joachim H. Ix
Manuscript ID: K360-2024-000146R2
Manuscript Title: Urine ammonium concentrations and cardiovascular and kidney outcomes in SPRINT participants with Chronic Kidney Disease
Date of Completion: June 20, 2024
Disclosure Updated Date: June 20, 2024

ASN American Society of Nephrology | JASN Journal of the American Society of Nephrology | KIDNEY360 Accessing Our World From Every Angle | CJASN Clinical Journal of the American Society of Nephrology

ASN Journal Disclosure Form

As per ASN journal policy, I have disclosed any financial relationships or commitments I have held in the past 36 months as included below. I have listed my Current Employer below to indicate there is a relationship requiring disclosure. If no relationship exists, my Current Employer is not listed.

R. Katz reports the following:
Employer: University of Washington

I understand that the information above will be published within the journal article, if accepted, and that failure to comply and/or to accurately and completely report the potential financial conflicts of interest could lead to the following: 1) Prior to publication, article rejection, or 2) Post-publication, sanctions ranging from, but not limited to, issuing a correction, reporting the inaccurate information to the authors' institution, banning authors from submitting work to ASN journals for varying lengths of time, and/or retraction of the published work.

Name: Ronit Katz
Manuscript ID: K360-2024-000146R1
Manuscript Title: Urine ammonium concentrations and cardiovascular and kidney outcomes in SPRINT participants with Chronic Kidney Disease
Date of Completion: April 18, 2024
Disclosure Updated Date: January 29, 2024

ASN American Society of Nephrology
JASN Journal of the American Society of Nephrology
KIDNEY360 Accessing Our World From Every Angle
CJASN Clinical Journal of the American Society of Nephrology

ASN Journal Disclosure Form

As per ASN journal policy, I have disclosed any financial relationships or commitments I have held in the past 36 months as included below. I have listed my Current Employer below to indicate there is a relationship requiring disclosure. If no relationship exists, my Current Employer is not listed.

K. Raphael has nothing to disclose.

I understand that the information above will be published within the journal article, if accepted, and that failure to comply and/or to accurately and completely report the potential financial conflicts of interest could lead to the following: 1) Prior to publication, article rejection, or 2) Post-publication, sanctions ranging from, but not limited to, issuing a correction, reporting the inaccurate information to the authors' institution, banning authors from submitting work to ASN journals for varying lengths of time, and/or retraction of the published work.

Name: Kalani L. Raphael
Manuscript ID: K360-2024-000146R1
Manuscript Title: Urine ammonium concentrations and cardiovascular and kidney outcomes in SPRINT participants with Chronic Kidney Disease
Date of Completion: April 27, 2024
Disclosure Updated Date: March 1, 2024

ASN
American Society of Nephrology
JASN
Journal of the American Society of Nephrology
KIDNEY360
Accessing Our World From Every Angle
CJASN
Clinical Journal of the American Society of Nephrology

ASN Journal Disclosure Form

As per ASN journal policy, I have disclosed any financial relationships or commitments I have held in the past 36 months as included below. I have listed my Current Employer below to indicate there is a relationship requiring disclosure. If no relationship exists, my Current Employer is not listed.

D. Rifkin reports the following:
Employer: VA San Diego and University of California, San Diego

I understand that the information above will be published within the journal article, if accepted, and that failure to comply and/or to accurately and completely report the potential financial conflicts of interest could lead to the following: 1) Prior to publication, article rejection, or 2) Post-publication, sanctions ranging from, but not limited to, issuing a correction, reporting the inaccurate information to the authors' institution, banning authors from submitting work to ASN journals for varying lengths of time, and/or retraction of the published work.

Name: Dena E. Rifkin
Manuscript ID: K360-2024-000146R2,
Manuscript Title: titled "Urine ammonium concentrations and cardiovascular and kidney outcomes in SPRINT participants with Chronic Kidney Disease,"
Date of Completion: June 20, 2024
Disclosure Updated Date: May 21, 2024

ASN American Society of Nephrology | JASN Journal of the American Society of Nephrology | KIDNEY360 Accessing Our World From Every Angle | CJASN Clinical Journal of the American Society of Nephrology

ASN Journal Disclosure Form

As per ASN journal policy, I have disclosed any financial relationships or commitments I have held in the past 36 months as included below. I have listed my Current Employer below to indicate there is a relationship requiring disclosure. If no relationship exists, my Current Employer is not listed.

J. Seegmiller reports the following:
Employer: University of Minnesota

I understand that the information above will be published within the journal article, if accepted, and that failure to comply and/or to accurately and completely report the potential financial conflicts of interest could lead to the following: 1) Prior to publication, article rejection, or 2) Post-publication, sanctions ranging from, but not limited to, issuing a correction, reporting the inaccurate information to the authors' institution, banning authors from submitting work to ASN journals for varying lengths of time, and/or retraction of the published work.

Name: Jesse C. Seegmiller
Manuscript ID: K360-2024-000146R1
Manuscript Title: Urine ammonium concentrations and cardiovascular and kidney outcomes in SPRINT participants with Chronic Kidney Disease
Date of Completion: April 18, 2024
Disclosure Updated Date: April 18, 2024

ASN American Society of Nephrology JASN Journal of the American Society of Nephrology KIDNEY360 Accessing Our World From Every Angle CJASN Clinical Journal of the American Society of Nephrology

ASN Journal Disclosure Form

As per ASN journal policy, I have disclosed any financial relationships or commitments I have held in the past 36 months as included below. I have listed my Current Employer below to indicate there is a relationship requiring disclosure. If no relationship exists, my Current Employer is not listed.

M. Shlipak reports the following:
Research Funding: Bayer Pharmaceuticals; Honoraria: Bayer; AstraZeneca; Boeringer Ingelheim;; Patents or Royalties: Kidney Health Monitoring in Hypertension Patients; Advisory or Leadership Role: American Journal of Kidney Disease; Journal of the American Society of Nephrology; Circulation; Board Member, Northern California Institute for Research and Education; and Other Interests or Relationships: Committee Member - KDIGO Guidelines Committee.

I understand that the information above will be published within the journal article, if accepted, and that failure to comply and/or to accurately and completely report the potential financial conflicts of interest could lead to the following: 1) Prior to publication, article rejection, or 2) Post-publication, sanctions ranging from, but not limited to, issuing a correction, reporting the inaccurate information to the authors' institution, banning authors from submitting work to ASN journals for varying lengths of time, and/or retraction of the published work.

Name: Michael Shlipak
Manuscript ID: K360-2024-000146R1
Manuscript Title: Urine ammonium concentrations and cardiovascular and kidney outcomes in SPRINT participants with Chronic Kidney Disease
Date of Completion: April 18, 2024
Disclosure Updated Date: May 8, 2023
