# Supplementary material for: Urine Ammonium Concentrations and Cardiovascular and Kidney Outcomes in Systolic Blood Pressure Intervention Trial Participants with CKD
Source: Kidney360. 2024 Jul 22;5(8):1077–86. doi: 10.34067/KID.0000000000000501 (PMC11371348; doi:10.34067/KID.0000000000000501)
Supplement: Supplementary file 2 [file kidney360-5-1077-s002.pdf]

Supplemental Table 1. Association of eGFR, urine ammonium and serum bicarbonate with baseline fasting status

	Baseline Fasting Status		
	**Yes**	**No**	**Missing**
N (%)	1943 (92.9)	103 (4.9)	46 (2.2)
eGFR (mL/min/1.73m^2)	49 (39, 55)	45 (37, 53)	47 (38, 55)
Urine ammonium (mmol/L)	14 (9, 23)	14 (9, 24)	15 (10, 21)
Serum bicarbonate (mmol/L)	26 (24, 27)	25 (23, 27)	26 (24, 28)

Supplemental Table 2. Correlation matrix of key variables relative to spot urine ammonium concentrations among SPRINT participants with CKD

	**NH_4/UCr**	**CO_2**	**eGFR**	**Urine ACR**
**NH_4/UCr**	1.000	-0.106*	0.112*	0.005
**Total Serum CO_2**		1.000	0.207*	-0.121*
**eGFR**			1.000	-0.288*
**Urine ACR**				1.000

Abbreviations: NH4, urine ammonium; UCr, urine creatinine

*Correlation is significant at the 0.01 level

Supplemental Table 3. Association of urine ammonium with composite CVD events and all-cause mortality in SPRINT participants stratified by total CO_2

Urine ammonium	N	# Events	Incidence rate (%/yr)	Model * HR (95% CI)	p for interaction
**CVD primary SPRINT event**					
Continuous (per two-fold higher), mmol/L					0.60
$CO_2 < 22$	149	20	3.77	1.81 (0.77, 4.22)	
$CO_2 \geq 22$	1943	235	3.38	1.23 (1.02, 1.49)	
**All-cause mortality**					
Continuous (per two-fold higher), mmol/L					0.24
$CO_2 < 22$	149	13	2.59	2.53 (0.74, 8.64)	
$CO_2 \geq 22$	1943	130	2.06	1.00 (0.77, 1.29)	

Abbreviations: CVD, cardiovascular disease; CO_2, serum total carbon dioxide; SBP, systolic blood pressure; DBP, diastolic blood pressure, ACEi, angiotensin-converting enzyme inhibitor; ARB, angiotensin-2 receptor blocker, HF, heart failure; BMI, body mass index; eGFR, estimated glomerular filtration rate; UACR, urine albumin-to-creatinine ratio

* Adjusted for age, sex, race, urine creatinine, randomization arm, SBP, DBP, number of antihypertensive meds, ACEi or ARB use, diuretic use, history of CVD or HF, tobacco use, BMI, LDL, total cholesterol, baseline eGFR and UACR

Supplemental Table 4. Association of baseline urine ammonium with primary CVD event and all-cause mortality in SPRINT participants by eGFR at baseline

Urine ammonium	N	# Events	Incidence rate (%/yr)	Model * HR (95% CI)	p for interaction
		**CVD primary SPRINT event**			
Continuous (per two-fold higher), ml/min/1.73m^2					0.87
eGFR < 45	814	86	3.03	1.13 (0.87, 1.47)	
eGFR ≥ 45	1278	57	1.23	1.39 (1.05, 1.83)	
		**All-cause mortality**			
Continuous (per two-fold higher), ml/min/1.73m^2					0.23
eGFR < 45	814	126	4.82	1.13 (0.84, 1.53)	
eGFR ≥ 45	1278	129	3.08	0.92 (0.61, 1.40)	

Abbreviations: CVD, cardiovascular disease; CO_2, serum total carbon dioxide; SBP, systolic blood pressure; DBP, diastolic blood pressure, ACEi, angiotensin-converting enzyme inhibitor; ARB, angiotensin-2 receptor blocker, HF, heart failure; BMI, body mass index; eGFR, estimated glomerular filtration rate; UACR, urine albumin-to-creatinine ratio

* Adjusted for age, sex, race, urine creatinine, randomization arm, SBP, DBP, number of antihypertensive meds, ACEi or ARB use, diuretic use, history of CVD or HF, tobacco use, BMI, LDL, total cholesterol and UACR

Supplemental Table 5. Association of urine ammonium with annualized eGFR change among SPRINT participants with CKD by randomization arm

	% annual eGFR change (95% CI)		
	Standard BP arm	Intensive BP arm	
Urine ammonium	N= 1035	N= 1057	p for interaction
Continuous (per two-fold higher)*	0.27 (-0.05, 0.58)	-0.19 (-0.55, 0.17)	0.41

Abbreviations: eGFR, estimated glomerular filtration rate; SBP, systolic blood pressure; DBP, diastolic blood pressure; ACEi, angiotensin-converting enzyme inhibitor; ARB, angiotensin-2 receptor blocker; CVD, cardiovascular disease, HF, heart failure

*Adjusted age, sex, race, urine creatinine, SBP, DBP, number of antihypertensive meds, ACEi or ARB use, diuretic use, history of CVD or HF, tobacco use, baseline serum potassium, baseline eGFR and urine albumin
